# Determination of Phosphate as an Ion-Association Complex of 11-Molybdovanadophosphate and Diindodicarbocyanine Based on Selective Oxidation of Excess Dye

**DOI:** 10.3390/molecules30091872

**Published:** 2025-04-22

**Authors:** Andriy B. Vishnikin, Svitlana V. Khlyntseva, Yaroslav Bazel, Ioseph Balogh, Ihor E. Barchiy

**Affiliations:** 1 Department of Analytical Chemistry, P.J. Šafárik University in Košice, 04154 Košice, Slovakia; khlyntseva@yahoo.com; 2 Department of Analytical Chemistry, Dnipro National University, 49050 Dnipro, Ukraine; 3 Department of Chemistry, University of Nyíregyháza, 4400 Nyíregyháza, Hungary; baloghj29@gmail.com; 4 Department of Inorganic Chemistry, Uzhhorod National University, 88000 Uzhhorod, Ukraine; igor.barchiy@uzhnu.edu.ua

**Keywords:** phosphate determination, polymethine dye oxidation, ion association complex, 11-molybdovanadophosphate, spectrophotometry

## Abstract

The elimination of absorbance of excess dye by selective oxidation was first proposed for analytical methods using the formation of ion-association complexes (IAs). On this basis, a new sensitive and selective spectrophotometric method for the determination of phosphate in the form of the IA of 11-molybdovanadophosphate with diindodicarbocyanine (DIDC) was developed. Symmetric diindodicarbocyanine and diindotricarbocyanine dyes can be completely oxidized by sufficiently strong oxidizing agents such as permanganate, dichromate, cerium (IV), and vanadate. Of the three dyes investigated (DIDC, N,N’-dipropyldiindodicarbocyanine, and diindotricarbocyanine), the best results were obtained with DIDC. A mixture of molybdate, vanadate, and nitric acid was preferably used as an oxidizing agent. Selective decolorization of only free dye ions, as well as changes in the IA spectrum compared to the dye spectrum, were explained by the isolation of the dye due to the formation of poorly soluble IA nanoparticles and changes in the redox potential of the dye due to its aggregation. The following optimal conditions for phosphate determination were found: 0.3 M nitric acid, 0.43 mM sodium molybdate, 0.041 mM sodium vanadate, 0.015 mM DIDC, and 18 min for the reaction time. The molar absorptivity of the IA was 1.86 × 10^5^ mol^−1^·L·cm^−1^ at 600 nm, and the detection limit for phosphate was 0.013 µM. The developed method was applied to the determination of phosphate in natural water samples.

## 1. Introduction

Phosphate is an essential substance for the growth and reproduction of living organisms and is a major factor in the characterization of rivers, lakes, and oceans [1,2,3]. A significant excess of the natural content of total phosphorus (>0.02 mg L^−1^) stimulates the growth of photosynthetic aquatic micro- and macroorganisms in undesirable amounts, which in turn leads to eutrophication of the water bodies and possibly to changes in the structure and functions of freshwater and marine ecosystems [4]. Several recent reviews provide a comprehensive overview of analytical methods for the determination of phosphate [1,5,6,7,8,9,10].

Spectrophotometric methods based on the reduction of the 12-molybdophosphate heteropolyanion (HPA) PMo_12_O_40_^3−^ (12-MPA) are most commonly used for the determination of orthophosphate [8,11,12,13]. The method exists in several variants, differing in the reducing agent used, acidity, and temperature. Standard spectrophotometric heteropolyblue-based procedures for phosphate determination have an LOD of about 10 μg L^−1^. This LOD is inadequate for the determination of dissolved reactive phosphorus in nutrient-poor (oligotrophic) waters [1,8]. Some of these methods are sensitive to high chloride concentrations due to a phenomenon known as salt error [11]. In contrast, procedures involving ion association complexes (IAs) can be performed in highly saline solutions.

An alternative method to increase the sensitivity of the spectrophotometric determination of phosphate is based on the formation IA between HPA and cationic dyes such as triphenylmethane, rhodamine, or polymethine dyes (PDs) [5,6,8,14,15,16]. IA-based methods are superior to heteropolyblue-based methods in several important respects, including about an order of magnitude higher sensitivity, rapidity, less dependence on the conditions of analysis (nature of the reducing agent, time, degree of reduction, and acidity of the solution), and higher selectivity toward chloride ions or transition metals.

Typically, the formation of IA results in the spectra of the dye and the IA being identical or only slightly different. In this respect, one of the main problems with approaches using IAs is how to remove excess dye. Previously, extraction and sorption on a suitable filter followed by dissolution in an organic solvent have been proposed to separate excess dye [17,18]. The use of organic solvents does not allow these methods to be classified as “green” methods of analytical chemistry. Additional stages complicate and lengthen the determination process. As a result, the achieved increase in sensitivity remains unattractive for most practical applications. In addition, partial co-extraction of the simple dye salt as well as impaired reproducibility limit the sensitivity. Some of these drawbacks can be eliminated or minimized using microextraction separation of unreacted dye [19].

One of the best existing methods for the determination of phosphate using IA is based on the conversion of the weakly colored protonated dicationic form of malachite green (MG) into an intensely colored single-charged form. The latter arises as a result of the formation of the IA of 12-MPA with the dye due to a shift in acid–base equilibrium [5,20,21,22]. The MG method is 3–4 times more sensitive than the molybdenum blue method and does not require a separation step. However, the formation of IA between triphenylmethane dye and HPA is slow, and the absorbance of the colored product can rise for many hours, especially in samples with low orthophosphate concentration [21]. Because of the use of micellar medium for IA solubilization and high acidity, not more than one or two MG cations are usually attached to 12-MPA [4]. Furthermore, because relatively high concentrations of molybdate and MG are used, a precipitate is formed in the mixed reagent solution, which must be filtered before the sample is analyzed. This requires preparation of the color reagent on the day of use and makes the method time-consuming [23].

Another extraction-less method utilizes changes in the dye spectra occurring during the formation of the IAs of HPAs with cationic dyes [16,24]. We have previously found that the reaction of sufficiently large and hydrophobic anions with cationic dyes is always accompanied by characteristic changes in the absorption spectra. This can be successfully used for the determination of the corresponding substances [25]. An important prerequisite for such reactions is a sufficiently low solubility of the resulting IA. It is worth noting that the same specific IAs are formed using the MG method.

Representatives of any cationic dyes, at least triphenylmethane, rhodamine, and polymethine dyes, as well as anionic dyes [26], can enter into reactions of such type. The reaction is very fast, simple, and direct. However, the sensitivity achieved was high but not maximum. Considering the molar absorptivities of the PDs reaching 3 × 10^5^ L mol^−1^ cm^−1^ [27] and the attachment of 3–6 PD cations to HPA, the molar absorptivity of IA and sensitivity of the method can be greatly improved. The working wavelength is usually chosen at the positive maximum of the difference spectrum of IA and dye. At this wavelength, the IA absorbs light significantly less than at the maximum of the dye spectrum. Some variants of this method use surfactants to prevent precipitation of IA [28], which can be a major source of sample contamination. Nevertheless, the use of surfactants is not mandatory. At IA concentrations less than 1 µM, the supersaturated phase of IA in solution is stable, and the absorbance remains unchanged for at least several hours.

The formation of IA nanoparticles can be used to develop other approaches. They are based on dye luminescence quenching and strong resonance scattering of incident light on IA nanoparticles [15,29,30,31]. The measurement of scattered light is poorly reproducible, especially at low concentrations, and the estimation of the small difference in fluorescence intensity required at the lower end of analyte concentrations is also subject to large fluctuations. These reasons reduce the attractiveness of these methods.

Automation of reactions using IAs faces difficulties associated with the sorption of sparingly soluble IA precipitate on the tube walls of the flow system. It has recently been shown that such difficulties can be minimized by using IA 12-heteropolymolybdates of phosphorus and silicon with the polymethine dye Astra Phloxine, provided that a sufficiently low concentration of the IA, less than 1 µM, is present in solution [16]. This allowed us to develop a highly sensitive and selective method for the consecutive determination of these elements.

**Figure 1:**
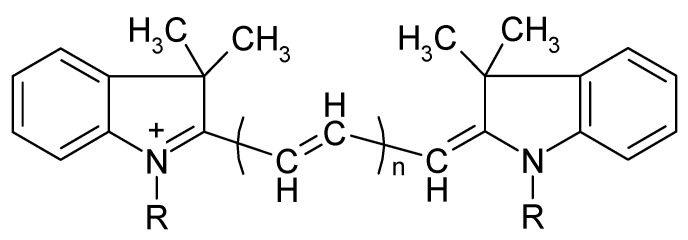
Chemical structure of polymethine dyes. DIDC—R = CH_3_, *n* = 2; Pr-DIDC—R = C_3_H_7_, *n* = 2; DITC—R = CH_3_, *n* = 3.

In this work, a new approach to eliminate the influence of dye excess based on the selective oxidation of excess PD ions that are not part of IA is proposed for analytical methods using IA formation. The applicability of this approach was demonstrated by the development of a simple, highly sensitive, and selective method for the determination of orthophosphate as an IA of 11-molybdovanadophosphate HPA (11-MVP) with PD. Three representatives of PDs were studied, namely diindodicarbocyanine (DIDC), N,N’-dipropyldiindodicarbocyanine (Pr-DIDC), and diindotricarbocyanine (DITC). The chemical structure of the dyes is shown in Figure 1. Chemometric optimization based on full factorial experimental design was used to find the optimum conditions. The developed method was applied to the determination of phosphate in water samples.

## 2. Results and Discussion

### 2.1. Investigation of Oxidation of Polymethine Dyes in the Presence of Heteropolyanion and Oxidant

In a preliminary study, it was established that symmetrical carbocyanine, dicarbocyanine, and tricarbocyanine dyes are capable of being oxidized under the action of sufficiently strong chemical oxidants. The reaction of such PDs with several oxidizing agents, namely potassium permanganate, potassium dichromate, cerium (IV) sulfate, potassium nitrate, potassium chlorate, potassium perchlorate, potassium iodate, potassium persulfate, potassium periodate, potassium bromate, sodium nitrite, and sodium vanadate, was investigated. The reaction was carried out in the presence of phosphate and molybdate. The acidity was chosen to be optimal for the formation of HPA. Under these conditions, the formation of isopolyanions and further formation of IAs with PDs can be excluded. It was found that phosphate, molybdate, and 12-molybdophosphate do not oxidize PDs.

**Figure 2:**
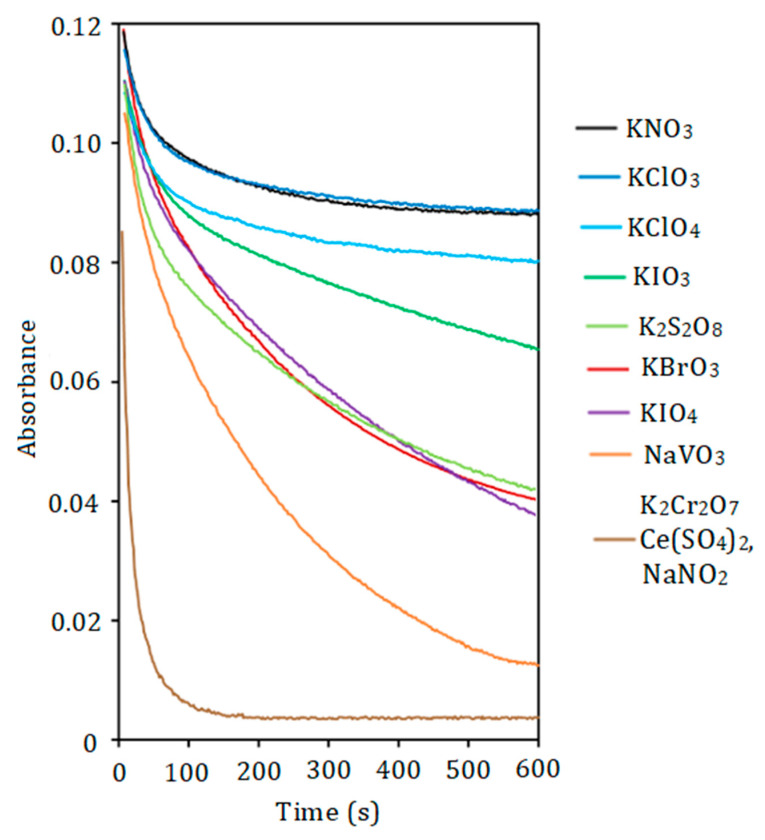
The effect of oxidant nature on the absorbance of DIDC. DIDC 1.0 µM, KH_2_PO_4_ 2.0 µM, Na_2_MoO_4_ 0.43 mM, oxidant 0.10 mM, HNO_3_ 0.22 M, *λ* = 636 nm, *l* = 1 cm.

The rate of PD oxidation correlates with the strength of the oxidant (Figure 2). Potassium dichromate, sodium nitrite, and cerium (IV) sulfate require 2–3 min to oxidize DIDC, whereas the reaction with permanganate occurs almost instantaneously. For the first three oxidizing agents, partial destruction of the dye in IA was observed. Permanganate completely oxidizes the dye ions that are part of the IA. Other oxidants studied oxidize DIDC too slowly. The use of an acidified mixture of molybdate and vanadate as both reagent and oxidant allows selective oxidation of only free dye ions not bound by HPA. Therefore, this mixture was chosen as the oxidizing agent. It was taken into account that IA in this case had the highest molar absorptivity, and the reproducibility of the results obtained with this reagent was the best. Also, compared to 12-molybdophosphate, mixed 11-MVP was found to be more stable in the strongly acidic medium used in the oxidation reaction studied. Other PDs including Astra Phloxine were stable to oxidation by most oxidizing agents tested. The mixture of Fe (II) and H_2_O_2_ used previously for the oxidation of DIDC [32] was excluded from consideration because H_2_O_2_ can degrade HPA, given the well-known ability of molybdenym (VI) and vanadium (V) to form stable peroxo complexes.

**Figure 3:**
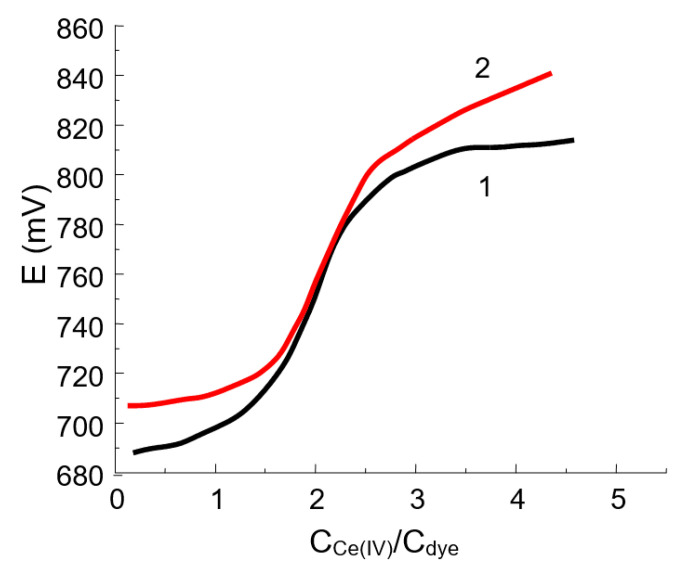
Titration curves for 10 mL of 0.112 mM DIDC (1) and 10 mL of 0.134 mM Pr-DIDC (2) with 1 mM cerium (IV) sulfate.

For all of the oxidants studied, the product of the reaction is yellow in color, indicating that the chromophore chain remains long enough. The curve of potentiometric titration of DIDC with potassium dichromate or cerium (IV) sulfate (Figure 3) has an inflection point corresponding to the fact that the dye loses two electrons. The following reaction scheme based on hydroxylation by the oxidation of DIDC or Pr-DIDC is proposed:

R-CH = CH-CH = CH-CH = R + 2[O] − 2e → R-CHOH-CHOH-CH = CH-CH = R

The oxidation of polyenes by various oxidants catalyzed by high-valent (d^0^) transition metals (V (V), W (VI), and Mo (VI)) usually leads to the formation of epoxides under mild conditions and to diols in the presence of water [33]. Another oxidation scheme was proposed in [32] with the cleavage of the double bond in DIDC by using the Fenton reagent, which had a high oxidation potential of 2.0–2.8 V and is characterized by the generation of a hydroxyl radical. The data obtained in the electrochemical oxidation of PDs, in particular DIDC and DITC [34,35,36], allowed us to conclude that the radical dications formed during one-electron oxidation undergo irreversible dimerization at even-methine carbon atoms.

PDs are converted to the colorless form by protonation in a sufficiently acidic solution (Table 1). The range of existence of the cationic forms of DIDC and Pr-DIDC is wider than for DITC. While DIDC is stable up to 1 M acid, the protonation of DITC is considerable even at pH > 1, which makes it impossible the use of some oxidizing agents. At the same time, sufficiently high acidity is required for the formation of heteropolyanion, and the oxidizing power of the most commonly used reagents increases at low pH. The strength of the dyes used as reducing agents increases in the following order: DIDC ≅ Pr-DIDC < DITC. Only sufficiently strong oxidants having a standard oxidation-reduction potential of approximately 1.0 V or more are able to interact with DIDC or Pr-DIDC.

### 2.2. Changes in Absorption Spectra During the Reaction Between PDs and Vanadate

**Figure 4:**
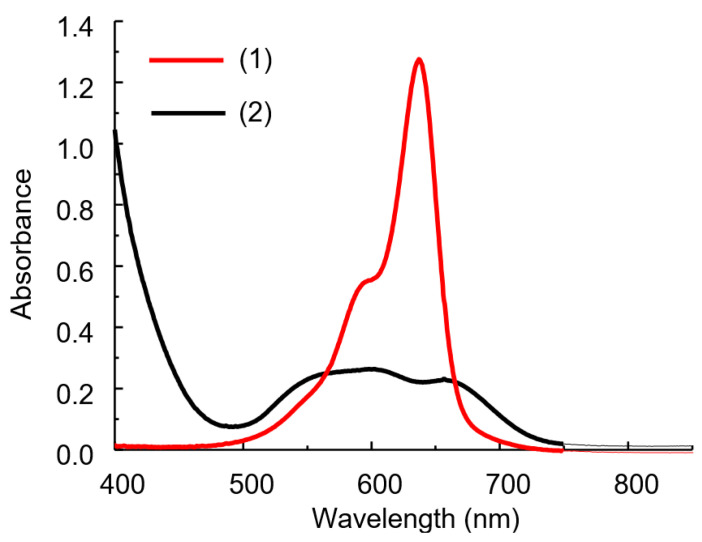
Absorption spectra of DIDC (1) and its IA with 11-MVP in the excess of HPA (2). DIDC 0.01 mM, KH_2_PO_4_ 0.1 mM, Na_2_MoO_4_ 0.43 mM, NaVO_3_ 0.041 mM, HNO_3_ 0.22 M, *l* = 1 cm.

**Figure 5:**
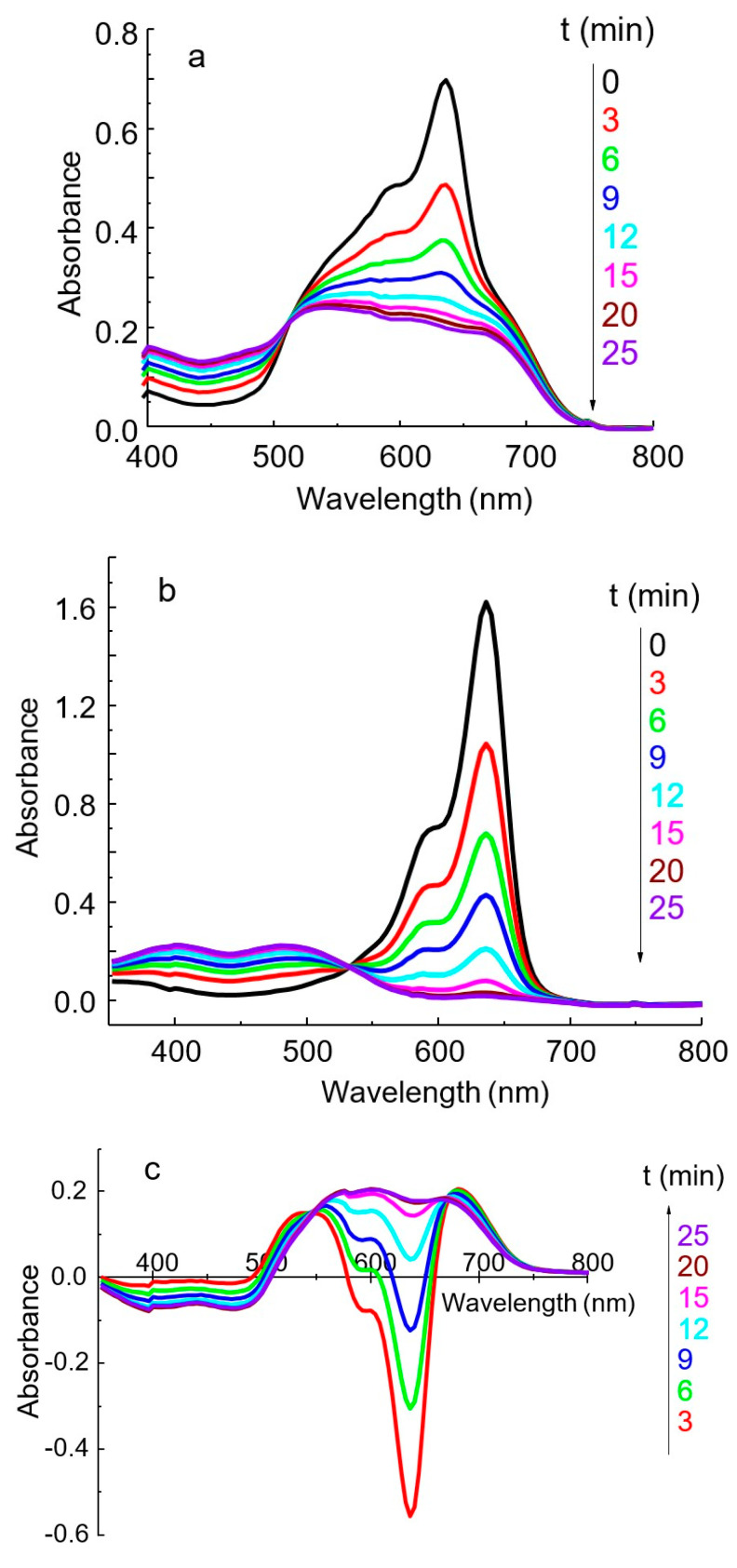
The effect of oxidation time on the absorption spectra of IA PVMo_11_O_40_^4−^ DIDC (**a**), reagent blank (**b**), and the difference between a–b (**c**). DIDC 0.015 mM, KH_2_PO_4_ 2.0 µM, Na_2_MoO_4_ 0.43 mM, NaVO_3_ 0.041 mM, HNO_3_ 0.22 M, *l* = 1 cm.

The IA spectrum differs significantly from the dye spectrum as a result of the reaction between HPA and DIDC (Figure 4) [16,24]. The solution spectra of the system under study represent the sum of the spectra of the oxidized and reduced forms of the dye, as well as the IA spectrum of DIDC with HPA. After 15–20 min, the oxidation of the excess dye is complete, and only IA remains in the reaction mixture. The spectrum of IA or actually aggregated dye consists of several strongly overlapping bands and has no pronounced maximum (Figure 4 and Figure 5a). As can be seen particularly well from the difference spectrum (Figure 5c), the dye band at 636 nm completely disappears. Instead, two bands appear in the spectrum at approximately 540 and 680 nm, shifted hypso- and batochromically with respect to the main dye band (Figure 5c). It is worth noting that a completely similar IA spectrum can be obtained without oxidation under conditions of large excess of counterion. The DIDC spectrum obtained in a tenfold excess of 11-MVP (Figure 4) contains the same three bands that were observed in the IA spectrum remaining after oxidation of the dye excess.

In the spectrum of the dye after oxidation, the band at 636 nm disappears, whereas two new bands appear at 400 and 490 nm (Figure 5b). Two bands with a similar position were observed in the spectrum of the PD obtained by electrochemical oxidation [34].

During the first ten minutes, a large negative difference is observed in the spectrum obtained by subtracting the spectrum of the blank solution from the IA spectrum (Figure 5c). The analytical signal corresponding to the dye maximum at 636 nm is high, but it cannot be measured with adequate reproducibility due to the very rapid oxidation of the dye. However, the absorbance measured at 600 nm becomes constant after 15–20 min, is easily reproducible, and is linearly related to the phosphate concentration. The isobestic point, present in all systems studied (Figure 5), indicates the interconversion of the reduced and oxidized forms of free dye ions. At the same time, the spectrum of the IA remains unaltered due to the rapid formation of this complex.

### 2.3. The Theoretical Background of Studied Reactions

The theory of self-aggregation of organic dyes can satisfactorily explain both the changes in the absorption spectra of PDs resulting from the formation of IA with 12-MPC and the selective oxidation of free dye ions. Self-aggregation of organic molecules has attracted interest in recent years in both fundamental and applied fields due to its novel chemical, physical, and optical properties. Self-aggregation of dyes in solution is a frequently occurring phenomenon in the chemistry of organic dyes due to the strong π–π dispersion interaction between the conjugated system of π-electrons in these molecules [27,40,41,42,43,44]. In the reactions studied in this work, conditions were created for forced convergence of dye cations as a result of the formation of sparsely soluble nanodispersed particles of IA. In this case, the π–π-dispersion interaction of the chromophoric–aromatic system of dyes increases, which, in accordance with the exciton theory, leads to the splitting of the excited electron level into two levels with lower and higher energy. The transition of electrons to these levels results in the appearance of one or two hypso- and/or bathochromically shifted bands in the absorption spectrum relative to the main dye band.

Aggregation of dyes in aqueous solution can be promoted by a number of different factors, starting with the well-known self-association phenomenon that occurs with increasing dye concentration. Aggregation can be also enhanced by sorption of the dye on the surface of crystals such as AgCl [45] or by binding to neighboring charged sites on polyelectrolyte molecules [46]. High concentrations of inorganic salts can have a significant effect on the formation of PD aggregates, which is explained by the decreased solubility of the formed IAs in concentrated salt solutions [47,48].

We have previously shown that a stable supersaturated solution of IA can be formed during the reaction between PD and HPA [16] or other large anions such as BiI_4_^−^ [25] due to the very low solubility of such IAs. The close contact between the molecules of IA in poorly soluble precipitate particles favors the noncovalent interaction of the PD cations with each other.

To explain the difference in the oxidation rate between free dye ions and those bound into IA, it can be assumed that the dye ions in IA are more or less isolated from contact with the oxidant in these aggregates, which strongly slows down their rate of oxidation. However, another hypothesis seems more reasonable. It is well known that aggregation can cause a change in the formal redox potential for the dye. The oxidation potential of the one-electron transition for the J-aggregate of 3,8:3,12-dimethylene-9,11-neopentylenethiadicarbocyanine-p-toluenesulfonate was 74 mV more positive compared to the similar value for the monomer [49]. In contrast to these data, the redox potential of the oligostreptocyanin dimer was shifted to the negative potential region by 0.22 V relative to the monomer from 0.98 V to 0.76 V [50]. In [51], the redox potential of thiacarbocyanine PD was shown to be shifted to the negative potential region from 0.96 V for the monomer to 0.78 V for the J-aggregate.

Thus, depending on the nature of the dye and the environment, different directionality of the oxidation potential during aggregation can be observed. In our case, aggregation leads to an increase in the oxidation potential of the dye contained in IA. Consequently, free dye ions, which have a more negative redox potential, react more easily with oxidizing agents. These data explain the stability of PD ions in the composition of IA with HPA by weakening of their reducing properties due to the formation of aggregates.

As has been shown in a number of articles [49,52], the oxidation potentials of PDs strongly decrease with increasing length of the polymethine chain. The data we obtained confirm this observation (Table 1). When selecting the optimal oxidant for the reaction under study, the highest selectivity for oxidation of only unbound dye ions will be observed if the redox potential of the oxidant used is between the redox potentials for free and IA-bound dye. Presumably, this range is between 0.7 (IA-bound dye) and 0.9 V (free dye). The standard redox potentials for the studied oxidants increase in the series 1.004 V (VO_2_^+^/VO^2+^) < 1.232 V (Cr_2_O_7_^2−^/2Cr^3+^) < 1.44 V (Ce^4+^/Ce^3+^) < 1.51 V (MnO_4_^−^/Mn^2+^). As can be seen from this series, the best agreement with the put forward assumption is observed for the vanadate system, which also explains the observed difference in the rate and completeness of dye oxidation in IA particles and in solution.

### 2.4. Optimization of the Conditions for the Determination of Orthophosphate

The optimal conditions for the formation of 11-MVP are well known [53]. In all experiments, the molar ratio of Mo to V was fixed at 11:1, and the concentrations of molybdate and vanadate were taken as 0.43 mM and 0.041 mM, respectively.

**Figure 6:**
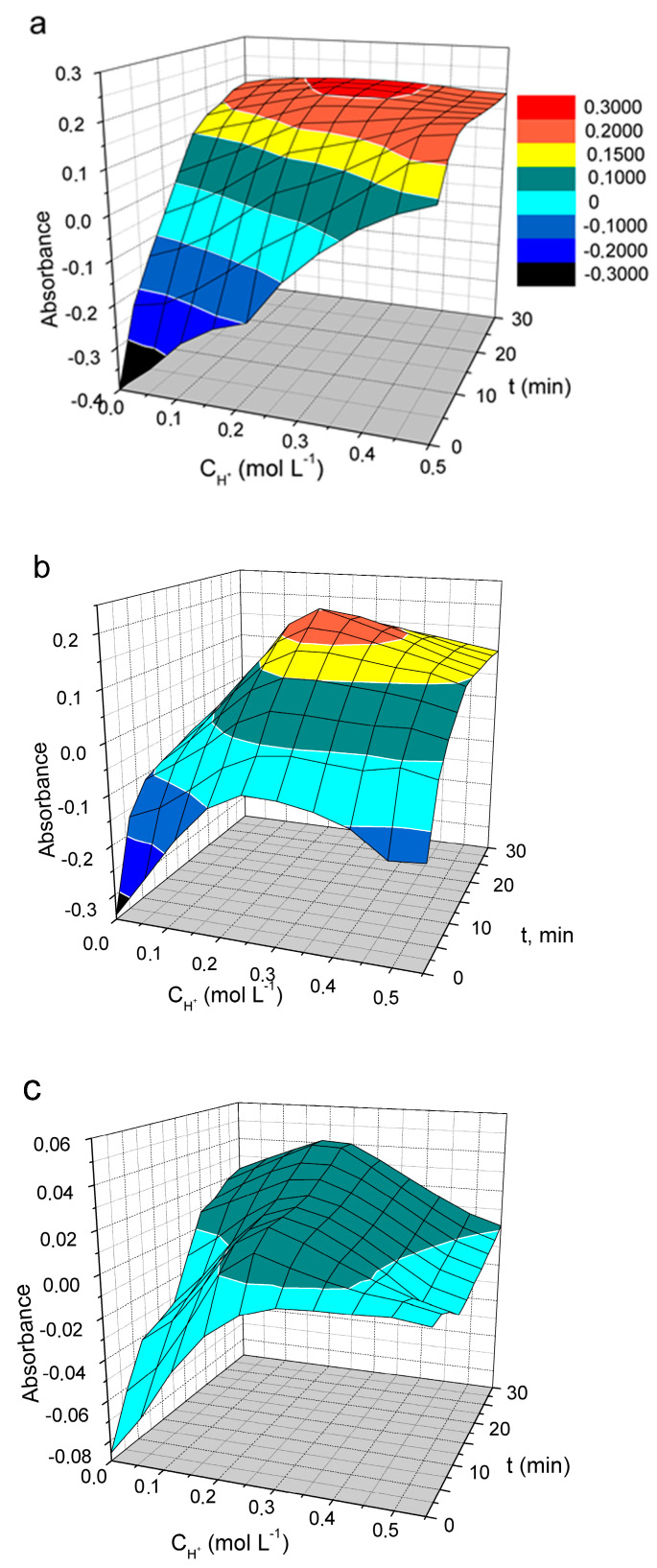
The effect of acid concentration and time of oxidation on the absorbance of IA DIDC-MVP (**a**), Pr-DIDC-MVP (**b**), and (**c**) DITC-MVP. Conditions: 0.43 mM Na_2_MoO_4_, 0.041 mM NaVO_3_, 2 µM KH_2_PO_4_, 0.015 M PD, *l* = 1 cm, *λ* = 600 nm (DIDC); *λ* = 630 nm (Pr-DIDC); *λ* = 640 nm (DITC).

The oxidation of PDs with vanadate is a slow reaction. The measured absorbance is highly dependent on the reaction time, the acidity used, and the dye concentration. The effect of these factors on the oxidation of DIDC, Pr-DIDC, and DITC by vanadate was studied using two-factor optimization and a response surface study. Optimal conditions for phosphate determination can be selected by studying the response surface since the three-dimensional picture gives a complete overview of the system. Because the number of significant factors in the studied system was more than two, one of the factors should be excluded to visualize the response surface. At the 95% confidence level (*p* < 0.05), weaker significance was observed for the dye concentration variable. Therefore, this factor was set at 0.015 mM as a compromise between the maximum response and acceptable absorbance of the blank solution. The effect of reaction time and acidity on IA absorbance is presented in Figure 6a.

The most suitable results were obtained by using DIDC because it was the only dye for which the absorbance remained constant over the wide range of variables studied. Only a maximum instead of a plateau was observed on the response surface for Pr-DIDC (Figure 6b). During storage, DITC is oxidized by oxygen dissolved in water. Therefore, it was not surprising that in this case, even the dye included in the IA was oxidized by the oxidant used, leading to strong variations and low values in the absorbance (Figure 6c). DIDC was selected to further optimize the optimal conditions for the determination of phosphate.

At the same time, the influence of the third factor on absorbance, namely the concentration of the dye, is significant. Thus, all three factors were found to be interdependent. In this case, a common option for finding optimal conditions is optimization by developing an empirical model of the response surface by the full factorial design.

### 2.5. Search of the Optimal Conditions by the Full Factorial Design

Optimization procedures are usually based on univariate optimization, which facilitates the interpretation of the results obtained but does not take into consideration the interactions between variables. Consequently, a false maximum may be attained, leading to the use of certain conditions under which the combination of variables does not provide the best analytical response. One commonly used approach of multivariate tools is the two-level full factorial design. However, preliminary calculations using this empirical model, as well as inspection of experimental response surfaces (Figure 6), show that second-order effects on the response must be accounted for.

The number of trials to be conducted in the 33 factorial design was reduced by using a central composite design. It consisted of a 2^k^ (k = 3, number of factors) factorial design, which provided data for estimating the first-order effects for each factor and the interactions between the factors and a “star” design consisting of 2k + 1 points that provided data to estimate second-order effects. Three factors, (1) dye concentration, (2) acid concentration, and (3) reaction time, could be systematically varied and optimized in the studied system (Table S1). A detailed description of chemometric optimization by full factorial design can be found in Supplementary Material.

**Figure 7:**
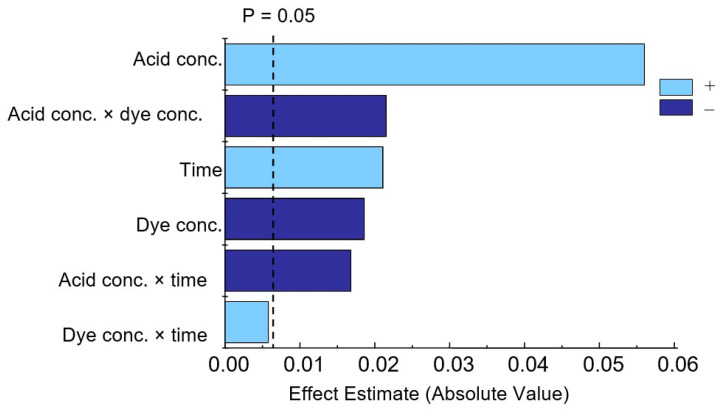
Pareto chart of standardized effects for variables in phosphate determination.

The effects of individual factors and their combinations were determined (Table S2). According to these studies, all three effects studied are significant, with acid concentration having the greatest influence. The interactions (acid concentration × dye concentration) and (acid concentration × time) are also statistically significant. In the Pareto chart (Figure 7), positive values represented by time and acid concentration indicate that upon their increase, the response also increases, while a negative value for dye concentration means that this factor has the opposite effect (Table S3).

In summary, the optimal conditions for spectrophotometric determination of phosphate with IA DIDC-MVP are 0.3 M nitric acid, 0.015 mM DIDC, and 18 min of reaction time. These values agree well with two-factor optimization results and the experimentally obtained response surface (Figure S1).

## 3. Analytical Performance

### 3.1. Method Validation

Based on the found optimal conditions, a spectrophotometric procedure for the determination of orthophosphate was developed. Its analytical characteristics are summarized in Table 2. The limit of detection (LOD) and limit of quantification (LOQ) were calculated according to the equations LOD = 3 × S_a_/b and LOQ = 10 × S_a_/b, where S_a_ is the standard deviation of the regression. The molar absorptivity of IA DIDC-MVP obtained from the slope was equal to 1.85 × 10^5^ mol^−1^ L cm^−1^.

### 3.2. Evaluation of Interference

The effects of potential interferences from ions that commonly accompany phosphorus (V) in natural and wastewater were investigated by the determination of 0.4 µM orthophosphate according to the recommended procedure. These species were largely tolerated without any pre-treatment. An ion was considered not to interfere if a relative error caused by it was less than 5%. The maximum tolerable concentrations of 23 foreign ions are given in Table 3 and, in general, significantly exceed the reported concentration levels of common pollutants in natural waters [12]. This indicates the high selectivity of the proposed method. However, AsO_4_^3−^, Fe^3+^, and WO_4_^2−^ ions introduce serious interferences to phosphate at molar ratios of 20:1 and 15:1, respectively. Silicate affects the determination of phosphate starting from 0.2 mM.

### 3.3. Analytical Application

The developed method was applied to the determination of phosphate in deep-well waters. The results obtained by the proposed method, the method using the formation of IA between Astra Phloxine and 12-molybdophosphate, and the standard method are presented in Table 4. The confidence intervals of the results of water analysis obtained by all three methods overlap each other, which confirms the accuracy of the developed method.

### 3.4. Comparison of the Present Method with Previous Methods for Orthophosphate Determination

**Figure 8:**
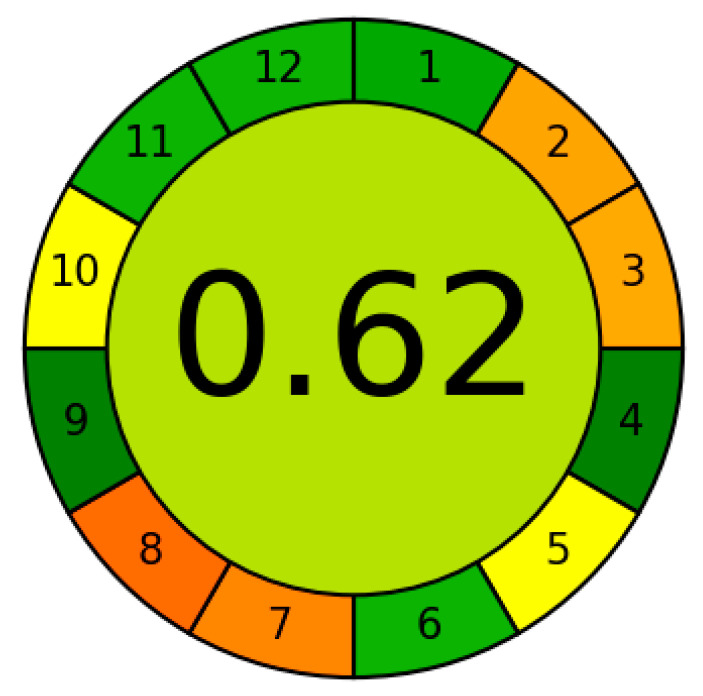
Assessment of the greenness of the proposed method by AGREE metric.

A comparison of the different methods developed for the determination of orthophosphate from 2019 to the present is presented in Table 5. Spectrophotometric methods based on the reduction of12-MPA by ascorbic acid and the formation of heteropolyblue remain in the spotlight. They are often combined with modern preconcentration methods and automated with flow methods of analysis. IAs are also used as analytical forms in combination with three detection methods, namely UV–Vis spectrophotometry, resonance Rayleigh scattering, and fluorescence. In terms of sensitivity, the method developed in this work competes confidently with all recently proposed methods. The advantage over methods based on the formation of heteropolyblue is due to the much higher molar absorption coefficient of IA DIDC-12-MPA, almost an order of magnitude higher. For heteropolyblue, it ranges from 15,000 to 24,000 mol^−1^ L cm^−1^ depending on the method. For the same reason, the sensitivity of the present method is superior or equal to methods using UV–Vis detection and preconcentration. The absolute limit of detection of most electrochemical, chromatographic, and atomic spectroscopic methods is also lower than that of the proposed method, although it should be noted that this table does not include methods developed before 2019. The greenness of the developed method was evaluated using the Analytical Greenness (AGREE) metric (Figure 8). The obtained score of 0.62 confirms its sufficiently high environmental friendliness.

## 4. Materials and Methods

### 4.1. Reagents and Apparatus

All chemicals used were of analytical-grade quality. Double distilled water was used throughout this experiment. A stock solution of phosphorus (V) containing 0.1 M phosphate was prepared by dissolving KH_2_PO_4_ in water. A 0.1 M molybdate solution was prepared by dissolving Na_2_MoO_4_ × 2H_2_O recrystallized from water–ethanol mixture, standardized by evaporation, weighed, and stored in a polyethylene bottle. DIDC iodide was purchased from Sigma-Aldrich, and the Pr-DIDC iodide and DITC fluoroborate were obtained as a gift from Prof. A.A. Ishchenko (Institute of Organic Chemistry, Kyiv, Ukraine).

A UV 4 UV–Vis spectrophotometer (Unicam, Great Britain) controlled by Software Vision 32-bit Version 1.10 was used to measure the absorption spectra. Glass cuvettes with path lengths of 1 and 5 cm were used.

Minitab Statistical Software 16.2.2. was used to perform statistical design of experiments.

### 4.2. Preparation of the Combined Reagent Solution

A sample of NaVO_3_ × 2H_2_O weighing 0.065 g was dissolved in 5 mL of hot water; after cooling, 12.5 mL of concentrated HNO_3_ was added, then 1.05 g of Na_2_MoO_4_ × 2H_2_O, and the solution was filled with distilled water to 100 mL. This solution was diluted five times before analysis. The final solution contained 8.6 mM molybdate, 0.3 M nitric acid, and 0.82 mM vanadate.

### 4.3. Procedure for Orthophosphate Determination

A sample containing from 0.03 to 0.3 µg of orthophosphate was placed in a 25 mL volumetric flask. Then, 0.5 mL of the combined reagent solution, 1.5 mL of 2 M HNO_3_, and 0.75 mL of 0.2 mM DIDC were added, and the solution was diluted to 10 mL. Absorbance was measured at 600 nm after 18–20 min against the reagent blank using a 5 cm cuvette.

### 4.4. Analysis of Water Samples

Water samples were filtered through a 0.45 mm membrane filter to remove particulate matter and immediately analyzed. Next, the procedure for the determination of phosphate in real samples was performed as described in the calibration. At least five repeated measurements were taken for each sample.

## 5. Conclusions

A new approach has been proposed to eliminate the influence of dye excess in reactions that utilize the formation of an ion-association complex between a heteropolyanion and an organic dye. It is based on the selective oxidation of a polymethine dye with a suitable oxidizing agent. It is shown that symmetric diindodicarbocyanine and diindotricarbocyanine PDs can be oxidized by sufficiently strong oxidants. The selective decolorization of only free dye ions as well as the strong changes in the IA spectrum compared to the dye spectrum can be explained by the isolation of the dye in the sparingly soluble IA nanoparticles and the change in the formal redox potential of the aggregated dye. A mixture of vanadate and nitric acid was chosen as the oxidizing agent. Although this reaction is slower than the reaction with potassium dichromate or cerium (IV) sulfate, it is more reproducible and has a lower limit of detection. Of the three dyes investigated, including DIDC, Pr-DIDC, and DITC, the best results were obtained with DIDC. The presented method describes a new route towards an eco-friendly methodology that eliminates the use of organic solvents in ionic associate-based assay procedures. The developed method is simple, highly sensitive, and selective. Its applicability was successfully tested for the determination of phosphate in several types of natural waters.

## Figures and Tables

**Table 1:** Spectral, acid–base, and redox properties of DIDC, Pr-DIDC, and DITC as well as their IA with 11-MVP.

Parameter	DIDC	Pr-DIDC	DITC
λ_max_^dye^ (nm)	636	641	748
ε_dye_ (mol^−1^ L cm^−1^)	1.82 × 10^5^	1.75 × 10^5^	2.32 × 10^5^ [37]
pK_p_	−0.05 [38]	-	1.03 [38]
pK_h_	12.77 [39]	-	11.84 [38]
E^o^ (V) (vs. SHE) ^1^	0.918	0.933	0.700
E_1/2_^ox^/(vs. Fc^+^/Fc) ^2^	0.287 [35]	-	0.066 [35]
λ_max_^IA^ (nm)	600	630	640
ε_IA_ (mol^−1^ L cm^−1^)	1.86 × 10^5^	1.72 × 10^5^	0.42 × 10^5^

^1^ Standard hydrogen electrode. ^2^ Ferrocene oxidation potential.

**Table 2:** Characteristics of the proposed procedure and calibration curve.

Characteristics	Value
λ (nm)	600
Reaction time (min)	18
Path length (cm)	5
Calibration range (μM)	0.04–0.4
Correlation coefficient	0.9991
Intercept ± Δ	0.246 ± 0.012
Slope ± Δ	(9.3 ± 0.5) × 10^5^
LOD (μM)	0.013
LOQ (μM)	0.04
Molar absorptivity (mol^−1^ L cm^−1^)	1.86 × 10^5^

**Table 3:** Tolerance levels of foreign ions in the determination of 0.4 µM orthophosphate.

Foreign Species	Tolerance Level (mM)
SO_4_^2−^	225
Cl^−^, NH_4_^+^	100
Na^+^, Li^+^, K^+^, Ca^2+^, Br^−^	50
Mn^2+^, Mg^2+^	20
Ni^2+^	6
Zn^2+^	5
Co^2+^, Cu^2+^, CO_3_^2−^	2
I^−^	1
CH_3_COO^−^	0.4
SiO_3_^2−^	0.2
Pb^2+^	0.2
F^−^	0.08
AsO_4_^3−^	0.01
Fe^3+^	0.01
WO_4_^2−^	0.006

**Table 4:** Determination of phosphate in deep-well water.

Sample	Proposed Method		IA 12-Molybdophosphate-Astra Phloxine FF [24]		Molybdophosphate Blue [12]	
	(C ± Δ) ^1^ (µM)	RSD ^2^ (%)	(C ± Δ) (µM)	RSD (%)	(C ± Δ) (µM)	RSD (%)
Artesian water	6.1 ± 0.3	4	6.3 ± 0.3	4	6.3 ± 0.4	5
Mineral water “Morshinska”	1.11 ± 0.06	4	1.23 ± 0.08	5	1.07 ± 0.08	6
Mineral water “BonAqua”	0.42 ± 0.03	5	0.41 ± 0.02	4	<LOQ	-

^1^ Δ—confidence limit (*n* = 5, *p* = 0.95). ^2^ RSD—relative standard deviation.

**Table 5:** Comparison of methods proposed for the determination of orthophosphate in 2019–2025.

Method	Description of the Method	LOD (μg L^−1^)	Linear Range (μg L^−1^)	Ref.
Molecular spectroscopy methods				
UV–Vis	Formation of molybdenum blue by reduction in 12-MPA using bismuth (III) and antimony (III) as sensitizers	13	20–1800	[54]
UV–Vis	Formation of IA between malachite green and 12-MPA	7	Up to 300	[23]
RRS	Formation of IA between cationic methyl violet and 12-MPA	4	8–200	[15]
Chemical sensor	Portable pump/valve-free water quality sensor based on phosphomolybdenum blue method	12	-	[55]
PTLS	Formation of molybdenum blue by reduction in 12-MPA	4	11–1000	[56]
UV–Vis, RRS, FL	Formation of IA between Nile blue and 12-MPA	4.8, 4.9, 5.2	5–100	[57]
Flow methods of analysis				
SIA-UV–Vis	The molybdenum blue method using antimony and ascorbic acid	7.7	Up to 2000	[58]
Microfluidic SIA	A miniaturized microfluidic hydrodynamic sequential injection system based on molybdenum blue method	1.5	3–30	[59]
µPAD	Phosphomolybdenum blue method with antimony (III) as catalysator and ascorbic acid as reducing agent	1.5	5–100	[60]
FB-SP analyzer	Formation of molybdenum blue by reduction of 12-MPA	4	Up to 360	[61]
Combination of UV–Vis spectrophotometry with preconcentration method				
MEPS-UV–Vis	Extraction of molybdoantimonatophosphoric heteropoly blue with hydrophobic C18 sorbent packed into a MEPS, elution with 60 µL of acetonitrile	0.4	1.3–24.8	[62]
Indirect UV–Vis	Separation of 12-MPA by extraction with isoamyl acetate, re-extraction with into aqueous phase, and formation of complex between molybdenum (VI) and sulfonitrazo	10	20–430	[63]
CPE-UV–Vis	Room temperature CPE with Triton X-100 procedure based on the heteropoly blue formation	-	1.6–63	[64]
MME-UV–Vis	Extraction of heteropoly blue with supramolecular solvent (alkyl polyglucoside and carboxylic acid)	5	20–400	[65]
Electrochemical methods				
CV	CV via [Omim]_6_Mo_7_O_24_—carbon paste electrode	2.6	3–3 × 10^5^	[66]
APSE	Phosphate-selective electrode with a plasticized membrane that includes bis(2-ethylhexyl)tin (IV) dichloride as an ionophore	24	60–3000	[67]
Chromatography				
CZE	Determination of phosphate in seafood by CZE with indirect UV–Vis detection	1.1 mg kg^−1^	5–55 mg L^−1^	[68]
IC	Suppressed IC after perchloric acid deproteinization in milk	33	1000–60,000	[69]
Atomic spectrometry methods				
EDXRF	Ultrasonically assisted dispersive micro-solid phase extraction using lanthanum oxide supported on graphene oxide	0.4	2–300	[70]
LIBS	Laser-induced breakdown spectroscopy	100	-	[71]
UV–Vis	Formation of IA between DIDC and 11-MVP, oxidation of dye excess with vanadate	0.4	1.2–12	This study

UV–Vis—Ultraviolet–Visible spectrophotometry; RRS—resonance Rayleigh scattering; PTLS—photothermal lens spectrophotometry; FL—fluorescence; SIA—sequential injection analysis; µPAD—microfluidic paper-based sensor; FB-SP analyzer—flow-batch syringe-pump-based flow analyzer; MEPS—microextraction by packed sorbent; CPE—cloud point extraction; MME—micellar microextractio; CV—Cyclic voltammetry; APSE—amperometric phosphate-selective electrode; CZE—capillary zone electrophoresis; IC—ion chromatography; EDXRF—energy dispersive X-ray fluorescence spectrometry.

## Data Availability

The original contributions presented in the study are included in the article/supplementary material, further inquiries can be directed to the corresponding author/s.

## Supplementary Materials

The following supporting information can be downloaded at: https://www.mdpi.com/article/10.3390/molecules30091872/s1, Figure S1: Response surface for IA absorbance as a function of acid concentration and reaction time at a constant dye concentration of 0.015 mM; Description of multivariate optimization of conditions for the determination of orthophosphate using the formation of IA between 11-molybdovanadophosphate HPA with DIDC and oxidation of excess dye; Table S1: Factors and their levels for a factorial experiment; Table S2: Design matrix and response values in the 33 central composite factorial design; Table S3: Estimation of model parameters for constructing a response surface.
