# Supplementary material for: Determination of Phosphate as an Ion-Association Complex of 11-Molybdovanadophosphate and Diindodicarbocyanine Based on Selective Oxidation of Excess Dye
Source: Molecules. 2025 Apr 22;30(9):1872. doi: 10.3390/molecules30091872 (PMC12073579; doi:10.3390/molecules30091872)
Supplement: Supplementary file 1 [file molecules-30-01872-s001.zip › molecules-3547819-supplementary.pdf]

Supplementary Materials to the article of Vishnikin A.B., Khlyntseva S.V., Bazel Y., Balogh I., Barchiy I.E. "Determination of phosphate as an ion-association complex of 11-molybdovanadophosphate and diindodicarbocyanine based on preliminary selective oxidation of dye"

Multivariate Optimization of Conditions for the Determination of Orthophosphate Using the Formation of IA Between 11-molybdovanadophosphate HPA with DIDC and Oxidation of Excess Dye

In the studied system, the following three factors were optimized: (1) acid concentration, (2) dye concentration, (3) reaction time.

The system under study was described by a complete second-order polynomial equation for three factors:

$$Y = b_0+b_1X_1+b_2X_2+b_3X_3+b_{12}X_1X_2+b_{13}X_1X_3+b_{23}X_2X_3+b_{11}X_1^2+b_{22}X_2^2+b_{33}X_3^2 \quad (1)$$

Estimates of the regression coefficients in this equation are obtained using the following formulas:

$$b_0=\frac{1}{N}\sum_{j=1}^{N}y_j\ ;\quad b_i=\frac{\sum_{j=1}^{N}X_{ji}y_j}{\sum_{j=1}^{N}X_{ji}^2}\ ;\quad b_{im}=\frac{\sum_{j=1}^{N}X_{ji}X_{jm}y_j}{\sum_{j=1}^{N}(X_{ji}X_{jm})^2}\ (i\neq m),\quad b_{ii}=\frac{\sum_{j=1}^{N}X_{ji}{}^{*}y_j}{\sum_{j=1}^{N}(X_{ji}{}^{*})^2} \quad (2)$$

where i is the factor number, j is the experiment number, and the terms X_{ji}* refer to the "star" points and are calculated as

$$X_{ji}{}^{*}=X_{ji}^2-\frac{1}{N}\sum_{j=1}^{N}X_{ji}^2 \quad (3)$$

Table S1 shows the levels for each factor, which were selected based on the results of previous experiments. Table S2 shows the design matrix, experimental and calculated responses. Each experiment was repeated three times, and the response for the factorial experiment was the average optical density obtained for each point of the design.

Table S1. Factors and their levels for a factorial experiment.

Фактори		Level 1	Level 2	Level 3
Acid concentration, M	X_1	0.015	0.215	0.415
DIDC concentration, mM	X_2	0.01	0.015	0.02
Recation time, min	X_3	10	15	20

The necessary data for constructing the response surface were calculated using a complete polynomial equation of the second degree for two factors:

$$Y = b_0+b_1X_1+ b_3X_3+ b_{13}X_1X_3+b_{11}X_1^2+b_{33}X_3^2 \qquad (4)$$

where the coefficients b_i and X_{ji}* were calculated using equations (2, 3).

The obtained coefficients are given in Table S3, and the corresponding response surface is shown in Figure S1.

Table S2. Design matrix and response values in the 3^3 central composite factorial design

Run	Coded factor levels						Response	
	X_1	X_2	X_3	X_1^a	X_2^a	X_3^a	Y_{exp}^b	Y_{calc}^c
1	-1	-1	-1	0.3333	0.3333	0.3333	0.0140	-0.0185
2	1	-1	-1	0.3333	0.3333	0.3333	0.2030	0.1700
3	-1	1	-1	0.3333	0.3333	0.3333	0.0020	-0.0242
4	1	1	-1	0.3333	0.3333	0.3333	0.1170	0.0783
5	-1	-1	1	0.3333	0.3333	0.3333	0.0530	0.0457
6	1	-1	1	0.3333	0.3333	0.3333	0.1870	0.1672
7	-1	1	1	0.3333	0.3333	0.3333	0.0760	0.0630
8	1	1	1	0.3333	0.3333	0.3333	0.1120	0.0985
9	1	0	0	0.3333	-0.6667	-0.6667	0.1650	0.2134
10	-1	0	0	0.3333	-0.6667	-0.6667	0.0790	0.1014
11	0	1	0	-0.6667	0.3333	-0.6667	0.1350	0.1574
12	0	-1	0	-0.6667	0.3333	-0.6667	0.1710	0.1946
13	0	0	1	-0.6667	-0.6667	0.3333	0.2090	0.1950
14	0	0	-1	-0.6667	-0.6667	0.3333	0.0900	0.1528
15	0	0	0	-0.6667	-0.6667	-0.6667	0.1990	0.2174

[a] X – terms of equation (3) in the "star" points

[b] Y_{exp} – experimentally obtained absorbance – average of 3 measurements

[c] Y_{calc} – absorbance calculated by full factorial design

Table S3. Estimation of model parameters for constructing a response surface.

b_0	b_1	b_3	b_{13}	b_{11}	b_{33}
0,0188	0,0538	0,0377	-0,0208	-0,0458	-0,0183

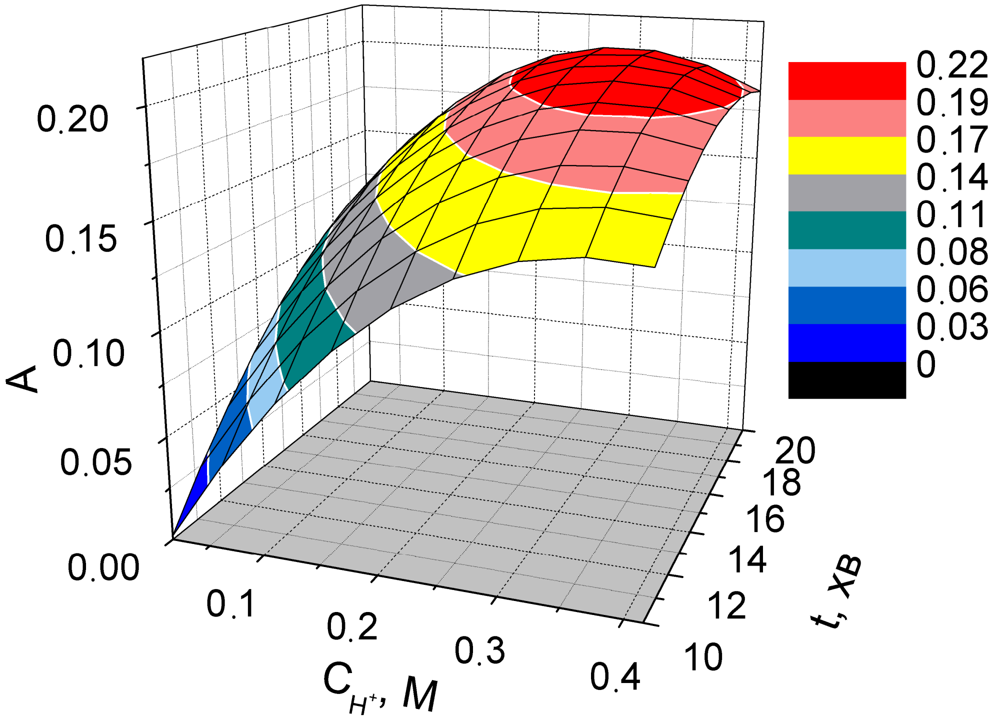

Figure S1. Response surface for IA absorbance as a function of acid concentration and reaction time at a constant dye concentration of 0.015 mM.
